# Resolving the infection process reveals striking differences in the contribution of environment, genetics and phylogeny to host-parasite interactions

**DOI:** 10.1186/1741-7007-9-11

**Published:** 2011-02-22

**Authors:** David Duneau, Pepijn Luijckx, Frida Ben-Ami, Christian Laforsch, Dieter Ebert

**Affiliations:** 1 University of Basel, Zoological Institute, Vesalgasse 1, Basel, Switzerland; 2 Department of Zoology, George S. Wise Faculty of Life Sciences, Tel Aviv University, Tel Aviv, Israel; 3 Department of Biology II and GeoBio-Center, Ludwig-Maximilians-University of Munich, Martinsried, Germany

## Abstract

**Background:**

Infection processes consist of a sequence of steps, each critical for the interaction between host and parasite. Studies of host-parasite interactions rarely take into account the fact that different steps might be influenced by different factors and might, therefore, make different contributions to shaping coevolution. We designed a new method using the *Daphnia magna - Pasteuria ramosa *system, one of the rare examples where coevolution has been documented, in order to resolve the steps of the infection and analyse the factors that influence each of them.

**Results:**

Using the transparent *Daphnia *hosts and fluorescently-labelled spores of the bacterium *P. ramosa*, we identified a sequence of infection steps: encounter between parasite and host; activation of parasite dormant spores; attachment of spores to the host; and parasite proliferation inside the host. The chances of encounter had been shown to depend on host genotype and environment. We tested the role of genetic and environmental factors in the newly described activation and attachment steps. Hosts of different genotypes, gender and species were all able to activate endospores of all parasite clones tested in different environments; suggesting that the activation cue is phylogenetically conserved. We next established that parasite attachment occurs onto the host oesophagus independently of host species, gender and environmental conditions. In contrast to spore activation, attachment depended strongly on the combination of host and parasite genotypes.

**Conclusions:**

Our results show that different steps are influenced by different factors. Host-type-independent spore activation suggests that this step can be ruled out as a major factor in *Daphnia*-*Pasteuria *coevolution. On the other hand, we show that the attachment step is crucial for the pronounced genetic specificities of this system. We suggest that this one step can explain host population structure and could be a key force behind coevolutionary cycles. We discuss how different steps can explain different aspects of the coevolutionary dynamics of the system: the properties of the attachment step, explaining the rapid evolution of infectivity and the properties of later parasite proliferation explaining the evolution of virulence. Our study underlines the importance of resolving the infection process in order to better understand host-parasite interactions.

## Background

Host-parasite coevolution is the result of multiple adaptations and counter-adaptations evolving in concert within the constraints of a particular system. Hosts use diverse defence mechanisms that coevolve with the offensive mechanisms of the parasite. From phages to ectoparasites, the success of infection depends on a series of steps and for each of them, the hosts may have specific defence mechanisms [1,2]. The following steps may be distinguished, with greater or fewer steps potentially existing depending on the system and the level of resolution: The host encounter with the parasite is the first step. During this step, the host may exhibit particular behaviours in order to avoid the parasite [3] and there may be polymorphism for such behaviours within species [4]. Once the encounter has taken place, parasites with a dormant stage may need to be activated in order to terminate diapause and initiate the infection process - for example, by endospore germination [see, for example, [5]]. After the activation step, endoparasites need to enter the host tissues. For many parasites, including the one studied here, this occurs through the attachment of the parasites to the host tissues but hosts may evolve to prevent this attachment. For example, plants often have very specific mechanisms to prevent fungal pathogens from entering leaf tissue [6] and some species produce layers upon their epithelium - the first barrier against infection - to obstruct parasite penetration (for example, mucus in coral protection [7] or salivary mucins to preserve the oral cavity health [8]). After attachment and entering its host, the next step of infection is proliferation. To counteract parasite growth, the host adapts physiologically (for example, iron-withholding [9]) or actively defends itself with an immune response. In the final step of infection, the parasite releases transmission stages to infect other hosts.

It has been argued that the fact that infection trials often intermingle the effects of different infection steps strongly influences our interpretation of host-parasite interactions [1,10,11]. For example, if only one of the steps is specific, the entire infection process will be specific. The same is true for environmental effects and host genotype-parasite genotype interactions. Furthermore, even if each of the steps is under simple genetic control (one or few loci) the combination of all of them might behave as a quantitative genetic trait and become more difficult to investigate. Resolving the infection process into its component steps simplifies the complexity of the infection process and helps us to better understand host-parasite interactions. Evolutionary models of host-parasite interactions are usually based on relatively simple assumptions about the underlying genetics and the impact of the environment. They commonly consider binary (Yes/No) infection outcomes (for example, matching-allele matrix [12-14]), even though available experimental data suggests more quantitative outcomes when looking at host and parasite interactions [15-17]. Explicit analysis of individual steps of infection can help bring in line theoretical models and data concerning the entire infection.

As little is known about the degree of specificity of the individual steps, the specificity attributed to host-parasite interactions is usually the combined effect of all steps. Although it is reasonable to assume that different steps are under the control of different genes, and are influenced by the environment to different degrees, it is possible that a single component of the infection pathway may explain most of the observed variation in host-parasite interactions. This is particularly important because understanding variation in host susceptibility is central for controlling disease and understanding evolution. Here, we use the *Daphnia*-*Pasteuria *host-parasite system to investigate which step(s) best explain(s) the high degree of host genotype by parasite genotype interactions reported for this system [18-20]. We analyse the contribution of host and parasite genetics, host gender, host phylogeny and of the environment for the dynamics of host-parasite co-evolution.

**Figure 1:**
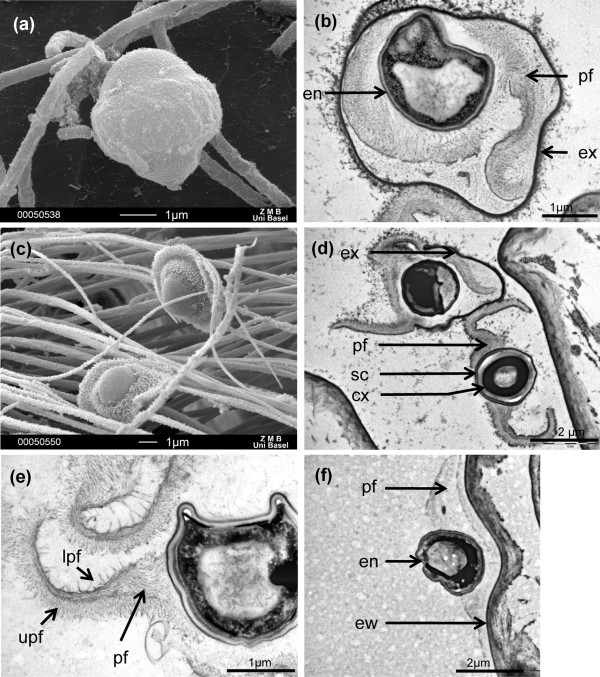
**Scanning electron microscopic (SEM) and transmission electron microscopic (TEM) images of the activation and the attachment step of the infection process of *Pasteuria ramosa *in *Daphnia magna***. (a) SEM image of a resting stage of *P. ramosa*. (b) TEM image of resting stage before activation. The exosporium (ex) encloses the two peripheral fibres (pf) and the endospore (en). (c) SEM image of activated spores trapped by *Daphnia *phyllopods. (d) TEM image of activated spores in *Daphnia *oesophagus. Top left: the spore is in the process of activating and shedding the exosporium. Bottom right: the activated spore with its sombrero-like structure in cross-section. Spore coat (sc) surrounding the cortex (cx). (e) TEM image of peripheral fibres (pf) and its microfibres on the upper side (upf) and on the lower side (lpf). The upper side is more furnished in microfibres and is more likely to play a role in the attachment. (f) TEM image of *Pasteuria *attached to the *Daphnia *oesophagus wall (ew). The nomenclature were defined according to the nomenclature of *Pasteuria penetrans *in [58].

Reproduction in planctonic crustacean *Daphnia *is primarily clonal, which is most suitable for dissociations of genetic and environmental effects of its interactions with parasites. *Daphnia *are frequently found to suffer from bacterial, fungal and microsporidial infections [21,22]; among them the Gram-positive bacterium *Pasteuria ramosa *[21-23]*. P. ramosa *produces endospores for transmission (Figure 1a and 1b[21]) that can remain dormant for decades [24]. Transmission is waterborne and endospores do not have flagella. The infection process is unknown but penetration of the host cuticula has been observed for the congeneric species *P. penetrans*, a parasite of root-knot nematodes [25]. Inside the host, *P. ramosa *proliferates in the haemocoel and musculature, castrates females and is transmitted horizontally after the release of endospores from the dead host [26,27]. The interaction of *D. magna *clones and *P. ramosa *clones has been shown to be specific [20]. *Pasteuria *was shown to impose strong selection on its host [28] and there is evidence for coevolution [29]. Furthermore, strong effects of the environment and genotype-environment interactions were reported for the overall infection process [30,31]. The goal of this study is to disentangle the different steps of the infection process and to analyse how they are shaped by host and parasite genetics, and the environment. We aim at finding the step that explains the greatest variance for the strong host-parasite interactions reported for the overall infection process.

We consider the following steps of the infection process and will investigate in details the second and the third, previously undescribed: (i) encounter; (ii) activation (once in contact with *Daphnia*, parasite endospores need a signal to germinate); (iii) attachment (the parasite must attach to the host and cross the host epithelium); (iv) proliferation (parasite proliferation and spore production); and (v) termination (killing the host to release spores). Environmental and host clone effects have been shown for the encounter and the proliferation steps [4,30,32-35]. However, neither of them can explain the strong host genotype by parasite genotype interactions described for the overall infection process in this system. Here, we localize where the activation and attachment steps take place and test for the genetic and environmental factors that influence those steps.

## Results

### Spore activation

We developed a new method that traces fluorescently-labelled spores of *P. ramosa *in the transparent *Daphnia magna *hosts in order to investigate the activation of parasite spores and the attachment of the parasite to the host. Within minutes of exposing the *Daphnia *host to the *P. ramosa *spores, we observed a characteristic change in spore morphology. Spores acquire a sombrero-like structure (Figure 1c and 1d) which corresponds to the shedding of the exosporium and the extension of the peripheral fibres. This morphology was never observed in spores not exposed to hosts. We call this morphological change in spore shape 'activation'. Activation was found to happen regardless of the host clone or *Pasteuria *clone used and was observed in both resistant and susceptible *D. magna *clones (Table 1).

**Table 1:** Results of infection trials, spore activation tests and attachment-tests.

			Infection trial			Spore activation			Attachment-test (attached out of five)		
			
Clones of *D. magna*		Pasteuria	C19	C1	C14	C19	C1	C14	C19	C1	C14
	Origin										
HO1	Hungary		R	R	R	Yes	Yes	Yes	0	0	0
HO2	Hungary		**S**	**S**	**S**	Yes	Yes	Yes	**5**	**5**	**5**
HO3	Hungary		R	R	R	Yes	Yes	Yes	0	0	0
M5	Belgium		R	R	R	Yes	Yes	Yes	0	0	0
M10	Belgium		**S**	**S**	**S**	Yes	Yes	Yes	**5**	**5**	**5**
Iinb1*	Germany*		R	R	R	Yes	Yes	Yes	0	0	0
Mu12	Germany		R	R	R	Yes	Yes	Yes	0	0	0
DG-1-106	Germany		**S**	R	R	Yes	Yes	Yes	**5**	0	0
AL144	Finland		R	**S**	**S**	Yes	Yes	Yes	0	**5**	**5**
Xinb3*	Finland*		**S**	R	R	Yes	Yes	Yes	**5**	0	0
XI*	Finland*		R	R	R	Yes	Yes	Yes	0	0	0
Xfa6*	Finland*		**S**	R	R	Yes	Yes	Yes	**5**	0	0
Kela-39-09	Finland		R	**S**	**S**	Yes	Yes	Yes	0	**5**	**5**
Kela-18-10	Finland		**S**	R	R	Yes	Yes	Yes	**5**	0	0

We tested all combinations of three *Pasteuria ramosa *clones (C19, C14, C1) with 14 *Daphnia magna *clones. Infection trials results are defined by exposing *Daphnia *to the parasites and determining the infection status after 20 days. Resistant means that none of the replicates were infected. Activation was determined by observing spores in the gut of the host with a sombrero-like shape. *R *means that the host clone is totally resistant to the concerned parasite clone. *S *means that the host clone is susceptible to the concerned parasite clone. *Yes *means that the spores were activated.

Labcross: 'Iinb1' is 'Mu11' (Belgium) selfed once; 'Xinb3' is 'X' (Finland) selfed three times; 'Xfa6' is 'AL144' selfed three times and crossed with 'Xinb3'; 'XI' is a cross between 'Iinb1' and 'Xinb3'.

### Spore attachment

**Figure 2:**
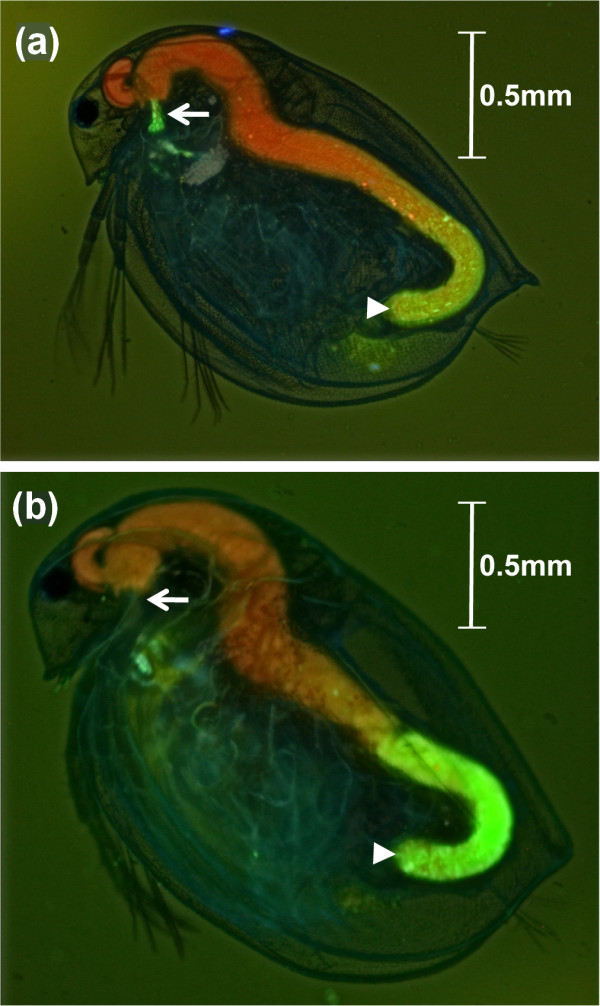
**Fluorescently labelled parasite spores attach to the oesophagus of susceptible, but not resistant, *Daphnia *clones**. (a) Picture of a susceptible *Daphnia magna *exposed to fluorescently labelled spores. The entire animal is shown. Parasites are attached on the epithelium of the oesophagus (arrow). Other labelled spores can be seen with the rest of the food in the end gut (arrowhead). (b) Picture of a resistant *D. magna *exposed to fluorescently labelled spores. The entire animal is shown. The oesophagus is free of parasite (arrow). Labelled spores can be seen with the rest of the food in the end gut (arrowhead). Note the autofluorescence of the mandibule. Extended focus images obtained by the camera Leica DFC 300FX and the program Leica Application Suite (Version 3.4.0, package 'Montage'). Intensity, contrast and sharpness were increased with the same strength.

We used different combinations of hosts and parasite clones previously characterized to be resistant or susceptible to given *Pasteuria *clones [20]. We observed the fate of fluorescent spores of three parasite clones exposed to 14 *D. magna *host clones with the aim of identifying differences which correlate with the compatibility of a given host-parasite combination (Table 1). The parasites attach to the host oesophagus for all susceptible (compatible) host-parasite combinations, but they never do so for the resistant combinations (Table 1, Figures 1f and 2a). Thus, the result of this attachment-test was 100% consistent with the results of infection trials (Table 1). For susceptible combinations the host oesophagus was densely covered with spores forming a dense layer in the oesophagus, while there were no spores attached in resistant combinations. We never observed ambiguous cases (for example, only few spores attached). While spores in the mid and end gut moved with the flow of the food, those attached to the oesophagus were not disturbed by passing boluses of food, indicating that they strongly adhere. In resistant combinations spores were never seen attached to the oesophagus and all spores passed with the flow of the food through the gut (Figure 2b). Thus, spore attachment in the oesophagus was very specific to the *D. magna *and *P. ramosa *genotype and consistent with resistant/susceptibility status for each combination.

### Influence of gender and culture conditions

Activation of spores was observed in all treatments and in all host clone-*Pasteuria *clone combinations (Table 2). In contrast, the specificity revealed by the attachment-test was found to be independent of host gender, temperature and culture conditions (single versus crowded; high versus low food, Table 2).

**Table 2:** Influence of the environment and host gender on spore attachment, as determined by the attachment-test.

		Treatments											
		
		LF, 20°C, single		HF, 20°C, single		HF, 10°C, single		HF, 15°C, single		HF, 25°C, single		HF, 20°C, crowded	
*Pasteuria*	*Daphnia*	Kela	Kela	Kela	Kela	Kela	Kela	Kela	Kela	Kela	Kela	Kela	Kela
clone	clone	39-09	18-10	39-09	18-10	39-09	18-10	39-09	18-10	39-09	18-10	39-09	18-10

C1	Female	**6/6**	0/5	**9/9**	0/9	**10/10**	0/10	**10/10**	0/10	**10/10**	0/10	**10/10**	0/10
	Male	**10/10**	0/10	**9/9**	0/9	**9/9**	0/10	**10/10**	0/10	**9/9**	0/10	**10/10**	0/10

C19	Female	0/10	**7/7**	0/8	**8/8**	0/9	**10/10**	0/10	**10/10**	0/9	**9/9**	0/10	**10/10**
	Male	0/5	**10/10**	0/10	**9/9**	0/10	**10/10**	0/10	**9/9**	0/9	**9/9**	0/10	**10/10**

Infection trials (see Table 1) showed that *Daphnia magna *clone Kela-39-09 is susceptible to *Pasteuria ramosa *clone C1 but resistant to C19. Kela-18-10 is resistant to C1, but susceptible to C19. LF = low food condition; HF = high food condition; single = *Daphnia *raised single in a 100 mL jar; crowded = *Daphnia *randomly picked from crowded cultures (high density). The bold characters highlights results where *P. ramosa *were attached to the *D. magna *oesophagus

### Spore activation and resistance of other *Daphnia *species

Spores were found to be activated after exposure to all *Daphnia *species tested (Table 3). We found that spores of the *P. ramosa *clone C19 were able to attach to the oesophagus and infect *D. dolichocephala *(Table 3) but did not attach to the oesophagus or infect *D. arenata*, *D. galeata*, *D. barbata*, *D. similis *or *D. lumholtzi*. We also tested other species for spore activation of *P. ramosa*. Upon exposure to *Simocephalus vetulus *(Daphniidae) spores were readily activated but did not attach to the oesophagus or infect any of the individuals tested. Upon exposure to mosquito larvae (*Culex *spp.), which are also filter-feeding but are not crustaceans, *P. ramosa *spores were neither activated nor attached to the host.

**Table 3:** Relationship between one *Daphnia magna*-derived clone of *Pasteuria ramosa *(clone C19) and several *Daphnia *species belonging to three different subgenera (*D. magna *belongs to the subgenus *Ctenodaphnia*).

Clones of *Daphnia *species	Sub-genus	Origin	Infection trial	Spore activation	Attachment-test (attached out of 5)
*D. arenata*	*Daphnia*	USA	R	Yes	0
*D. galeata*	*Hyalodaphnia*	Germany	R	Yes	0
*D. barbata*	*Ctenodaphnia*	Zimbabwe	R	Yes	0
*D. similis*	*Ctenodaphnia*	Israel	R	Yes	0
*D. lumholtzi*	*Ctenodaphnia*	Zimbabwe	R	Yes	0
*D. dolichocephala*	*Ctenodaphnia*	South Africa	S	Yes	4

Legend as in Table 1

## Discussion

The aim of the current study was to analyse two steps in the life cycle of a bacterial parasite, characterize the specificity of the interaction, with regard to genetic and environmental factors, and relate these findings to what is known about host-parasite coevolution in this system. We focused on the activation of the parasite's resting stages and on the attachment of the activated spores to the host tissue where it enters the host. Our study revealed that *P. ramosa *spores captured by the filter feeding *Daphnia *are indiscriminately activated by every *Daphnia *clone and *Daphnia *species tested (Tables 1 and 3). Furthermore, activation was not only found to be independent of the host genotype or species and host gender but also of the environmental conditions (namely, density, temperature and food conditions). The following step of the infection process, however, the attachment of the activated spore to the oesophagus wall of the host, depended strongly on the combination of the *D. magna *and parasite genotype, but not on the host's gender, nor the environmental conditions in which they were kept (Tables 1, 2, 3).

Previous studies with the *Daphnia-Pasteuria *system were not able to disentangle the activation, attachment and proliferation steps. Thus, variation in infection success as reported in earlier studies [19,32,35-40] may be explained by the combined effects of these steps. However, the binary polymorphism found in infection trials with high doses of single parasite clone [20] correlates perfectly with the results of our attachment-test (Table 1). This suggests that only *Pasteuria *clones able to attach to the oesophagus are able to infect the host. Ben-Ami *et al*. [38] proposed that *D. magna *might be either completely resistant or susceptible to *P. ramosa *depending on the genotype-genotype interaction. They called this the 'binary infection hypothesis'. Our data are consistent with this hypothesis and further pinpoint which specific step of the infection process is responsible for the high degree of specificity. For a given combination of host and parasite genotypes, the activated spores are either able to attach and then infect or they do not attach and do not infect. We did not see any evidence for a graded (quantitative) form of interaction.

### Spore attachment is a key step in *Daphnia-Pasteuria *coevolution

The *Daphnia-Pasteuria *system has become one of the prime examples of antagonistic coevolution. Host and parasites show strong genetic effects for resistance, virulence and infectivity; genotype × genotype interactions have been reported within and across populations and selection acts rapidly in natural populations [18,19,40,41]. Our study suggests that the parasite-dependent [28] host population structure and the coevolution [29] described for this system are mainly driven by the properties of a unique step, the attachment step. First, this step revealed very strong host genotype by parasite genotype interactions (Table 1). Second, the attachment step is independent of the environmental conditions. Third, a recent study of *D. magna - P. ramosa *coevolution using resurrected host and parasite isolates from lake sediments showed a signal of fluctuating selection only for infectivity but not for parasite virulence [29]. Virulence (the parasite's effect on infected hosts) was also observed to evolve but at a slower rate [29]. The authors proposed that the difference between the evolution of virulence and infectivity resulted from different genes contributing to these traits. Here we give a mechanistic explanation for this finding. Infectivity depends on the attachment and, most likely, on the ligands present on the host and on the parasite. On the other hand, expression of virulence may depend on the host's immune response during the within-host proliferation step. It is likely that these processes are determined by different sets of genes.

The identification of the attachment step as the key step in the coevolutionary dynamics in this system will allow us to improve our understanding of the patterns of antagonistic coevolution. For example, evolutionary models studying the coevolution of the infectivity and the virulence steps [42] can fit our system in relation to the coevolution of the attachment and the proliferation steps. Those models typically characterize infection outcomes as binary (Yes/No), while empirical data suggest they are more quantitative [15-17]. We show that we can observe a binary outcome when individual steps of the infection process are considered. Furthermore, our method provides a fast and reliable way to test individuals and populations for their susceptibility to *Pasteuria*. Ongoing research in our group showed that up to 400 *Daphnia *individuals can be tested in a day (P Luijckx, in preparation). The assay we developed makes it possible to test for susceptibility without the potentially confounding effect of the within-host proliferation step in the infection trials.

### From the environment to the host body cavity

The resting endospores of *P. ramosa *can remain dormant for decades under harsh environmental conditions [24,29]. Before attachment to the host, the spores need to be activated (Figure 1d). The filter-feeding *Daphnia *capture particles, including parasites, from the water and transport them on a mucus-layered pathway from the phyllopods to the mouth. During this process, the parasite's exosporium opens by an unknown trigger, releasing the activated spore form within less than 10 min (Figure 1). Despite the fact that spore activation is a necessary step for the infection, this step is entirely unspecific with regard to *Daphnia *species and clone, host gender and the environmental conditions (Tables 1, 2, 3). The signal that triggers spore activation may be related to chemical substances in the mucus of the filtering apparatus, but other factors (for example, mechanical) cannot be excluded.

Once the activated spore enters the oesophagus, it will attach to the oesophagus wall if the host and the parasite genotype are compatible. There it presumably penetrates the gut wall and enters the host's body cavity. A similar attachment process on the cuticula is also known from *P. penetrans *but, in this case, the parasite seems to be able to attach to any area of the nematode's body surface [25]. It has been proposed that the lower part of *P. nishizawae *attaches to the host because this part is densely covered by microfibres [43]. In contrast, in *P. ramosa *it is the upper part of the peripheral fibres (Figure 1e) that are most densely covered with a layer of microfibres. These fibres may be involved in the attachment (Figure 1f).

An endospore adhesin epitope, situated on the exosporium of *P. ramosa*, has been identified and it has been suggested that it may be a ligand responsible for the recognition and the binding onto the host [44]. However, according to our results, it is unlikely that this epitope is involved in the attachment because the exosporium of *P. ramosa *is removed during the activation step. A later study, analysing surface proteins of *P. ramosa *spores by two-dimensional gel electrophoresis, proposed that a collagen-like protein may be responsible for the binding onto the host but might suffer the same problem of the previous study [45]. We propose that later studies on candidate proteins responsible for the specific attachment to the host in this system should investigate the spores once activated.

The development of *Pasteuria*, from the moment they attach to the oesophagus until the vegetative stage can be detected in the hemolymph (about 8 days at 20°C [46]), is unknown. Also, the penetration mechanism is poorly described. Sayre and Wergin [25] show a transmission electron micrograph of *P. penetrans *with a structure they call a 'germ tube' crossing the host epithelium. Our hypothesis is that the endospore makes a hole across the host epithelium and injects its cortex into the host. As one response of *Daphnia *to wounding is an increase of Phenoloxidase (PO) activity [47], one might expect the penetration process to trigger an immune response but this remains an open question. However, resolving the infection process will allow the study of the immune response during the proliferation step without the confounding effect of genetic variation in the attachment step.

### Environment effects and the proliferation step

We found that environmental effects do not influence the activation and attachment step (Table 2). Excluding these steps, we suggest that the proliferation step is the one responsible for the reported sensitivity of the overall infection process for environment effects [32,34]. The activation and the attachment steps seem independent of the host's immune system (defined as a system that is potentially able to kill parasites), while the proliferation step is likely to be governed by the host's immune system. The immune system may lead to variation between and within those *Daphnia *clones that allow *Pasteuria *attachment (and, thus, enable the parasite to enter the host), thereby contributing to local and temporal adaptation, maternal effects and induced resistance [29,34,48]. We suggest that future studies on host immunity should use only *Pasteuria *clones that can attach to a given clone of *Daphnia *so that all the variation observed is likely to originate from variation during the proliferation step. These factors highlight the importance of controlling the host and parasite genotypes and breaking down the infection process in order to understand the respective role of each step in host-parasite interactions.

### Resolving the infection process leads to a better understanding of host-parasite interactions

Resolving the infection process in its sequential steps has been proposed in a number of theoretical models [10,11] but experimental data are scarce. Our approach is transferable to other host-parasite systems and our results suggest that this can provide important new insights into host-parasite interactions and their evolution. Increasing the degree of the resolution of the infection processes highlights a universe of possibilities of the different levels at which host and parasites interact. The different steps might differ in how they are influenced by the environment. They might also differ in which sets of genes regulate them. As it is probably the case for our study system, different steps of the infection process might follow distinct evolutionary dynamics and be explained by different model (for example, balancing selection, directional selection) [10,11]. However, because of the sequentiality of the steps, it is possible that the selection on one might depend on the selection on other steps. We propose that analysing infection as a succession of well characterized steps will help to reconcile the empirical data with predictions based on alternative coevolutionary models (for example, Red Queen and Selective Sweep models).

Spores of all *P. ramosa *clones tested, and which were isolated from natural *D. magna *populations, were activated by all *D. magna *clones as well as by six other *Daphnia *species (Table 3) and even a Cladoceran from a different genus, *Simocephalus vetulus*. Also, apart from the natural host, *D. magna*, *D. dolichocephala*, also became infected following attachment of the activated spores to the host oesophagus. This suggests that the triggers for spore activation and, to a lesser extent, for attachment are phylogenetically conserved. This may facilitate the host range evolution of the parasite. Indeed, despite its high specificity on the level of the host clone, *P. ramosa *infections have been reported in several species within the family Daphniidae [49]. It will be necessary to test more clones of different *Daphnia *species in order to determine their pattern of susceptibility and resistance to the parasite. Importantly, phylogenetically conserved steps of the infection process can be ruled out as major factors in coevolution, but are, perhaps, the most appropriate targets for vaccine and drug development. In fact, the genes involved in some infections steps have been worked out for some systems [50,51] and can be of use in biomedicine for diseases control [52,53].

## Conclusion

Our study highlights the explanatory power of resolving the steps of the infection process in order to better understand host-parasite interactions and coevolution. Attachment appears to be the crucial step for the previously observed high specificity in the *Daphnia-Pasteuria *system and we speculate that it is the crucial step for coevolution as observed in this system [29]. Our results reveal that each step can involve different interactions between host, parasite and environment and that certain steps can be phylogenetically conserved. With this knowledge, it will be easier to apply simple models of host-parasite interactions to this system and identify the mechanistic basis of trade-offs, maternal effects, genotype × environment interactions and coevolution. The logic of this procedure can equally be applied to other host-parasite systems but also to study other types of biotic interactions.

## Methods

### Host and parasite

We used 14 isofemale lines (hereafter referred as clones) of *D. magna *and one clone each of six other *Daphnia *species (Tables 1 and 3). Unless otherwise stated, *Daphnia *clones were kept in standard medium (ADaM, [54] modified by using only 5% of the recommended Selenium dioxide concentration) at 20°C and fed with the chemostat cultured unicellular algae, *Scenedesmus obliquus*.

The parasites used were single genotypes of *P. ramosa*, C1, C14 and C19, characterized as clones in Luijckx *et al*. [20] and originated from *D. magna *populations in Moscow (Russia), Tvärminne (Finland) and Gaarzerfeld (Germany), respectively. Spore suspensions of *Pasteuria *were obtained by homogenizing infected *D. magna *in ADaM and quantifying spore density. The status of resistant or susceptible *D. magna *were defined previously [20]. The infection status of two further Finnish *D. magna *clones ('Kela-39-09' and 'Kela-18-10') exposed to *Pasteuria *clones were tested with the same protocol. All infections in these experiments were done with naïve individuals born to naïve mothers, kept under high food conditions. These conditions were applied because they are known to minimize the triggering of immune effect [34,35,55].

### Fluorescence labelling of spores

Fluorescently labelled spores of *P. ramosa *were produced by homogenizing infected *Daphnia *in ADaM, followed by centrifugation at 10 000 g for 5 min at room temperature. The spore pellet was suspended in 0.5 mL of 0.1 M sodium bicarbonate (pH 9.1) containing 2.0 mg/mL of fluorescein-5(6)-isothiocyanate (F3651-100MG, Sigma-Aldrich, Miss, USA), a green fluorescent dye that stains proteins unspecifically [56]. Spores were incubated in the dark for 2 h at room temperature with occasional vortexing. The suspension was centrifuged at 10 000 g for 5 min and the supernatant removed. The spore pellet was suspended in distilled water and, again, subjected to centrifugation. This process was repeated until the supernatant was clear. Labelled spore suspensions can be stored at 4°C in the dark for several months.

The shape and location of the green labelled spores were examined in the transparent *Daphnia *using a microscope with fluorescent light (Leica DM 2500, at magnification 200 × and 400 ×) and filter cubes Leica B/G/R (bandpass filter excitation 420/30 nm; 495/15 nm; 570/20 nm - band pass filter suppression 465/20 nm; 530/30 nm; 640/40 nm). We increased the colour contrast by adding a red fluorescent dye to the medium in which the *Daphnia *were observed. This was done by preparing a solution of concentrated red dye (0.05 mL of DMSO with 0.0015 g of Tetramethylrhodamin-5-isothiocyanate; T0820-5MG by Sigma-Aldrich), which was homogenized in PBS to make the diluted dye (1 μL of concentrated solution with 10 mL of phosphate buffered saline). We added 1 μL of this solution to the *Daphnia *medium 10 min before observing the *Daphnia*. We obtained extended focus images using a camera Leica DFC 300FX and the program Leica Application Suite (Version 3.4.0, package 'Montage').

### The separation of the different steps and their specificity

Adult *Daphnia *were put individually in 1 mL of medium in 24-well-plates and exposed for at least 1 h to around 17,000 labelled *P. ramosa *spores. Susceptible hosts exposed to labelled spores become infected, suggesting that the dye does little or no harm to the spores (data not shown).

### Spore activation

Pilot trials revealed that the labelled spores remain in their typical spherical shape as long as they are not in contact with a host. Upon contact with the host phyllopods (swimming and respiratory appendages of branchiopod crustaceans), spores with a sombrero-like shape are observed (Figure 1c and 1d). We called this process 'spore activation'. We tested all combinations of 14 *D. magna *clones and three *P. ramosa *clones for spore activation (Table 1). The same was done for one clone each of six further *Daphnia *species but only in combination with one *P. ramosa *clone (Table 3). We used five replicates for each host-parasite combination [in total (14 × 3 × 5) + (6 × 1 × 5) = 190; details in Table 1].

### Spore attachment

After exposure, *Daphnia *were placed on a microscopic slide and we examined the complete *Daphnia *body under a fluorescent microscope. The transparent body of *Daphnia *allowed us to determine in which body region activated fluorescent spores attach in the living animals. Once we determined the specific area, we tested resistant and susceptible *Daphnia magna *clones (five replicates of 14 clones, details in Table 1) for differences in attachment. The same was done with clones of other *Daphnia *species (five replicates of one clone per species; details in Table 3). In order to validate the assignment of individuals with apparently no spores attached to their oesophagus, we viewed the oesophagus of slightly squashed animals at 400 × magnification. For each experiment, the examiner was not informed whether the animals belonged to a susceptible or to a resistant clone. In order to confirm that the *Daphnia *ingested spores, the gut content was inspected for the presence of spores. All exposed animals had spores in the faeces. We call this procedure to test for spore attachment the 'attachment-test'.

### Influence of gender and culture conditions

In order to see if the specificity pattern observed in the attachment-test was dependent on host sex or culture conditions, 10 host individuals of each sex were tested in each of six treatments. This was done with *D. magna *clones 'Kela-39-09' and 'Kela-18-10"'because these two *Daphnia *clones have the reverse pattern of infectivity to the two *P. ramosa *clones used and they are easily induced to produce male and female offspring in the laboratory. *Daphnia *were raised either at one of four temperatures (10°C, 15°C, 20°C, 25°C, with high food), two food levels (at 20°C, fed daily with 2.5 or 5 million algae) or two density levels (at 20°C, high food level, single *Daphnia *or *Daphnia *from crowded stock cultures; see Table 2). These conditions were chosen to represent various environments that are common in natural *Daphnia *populations. We did not employ a full factorial design, as our interest was not in establishing reaction norms but in testing for the influence of non-genetic conditions in general. *Daphnia *of both clones raised under these conditions were exposed to fluorescently labelled spores of *P. ramosa *C1 and C19. Given the very clear effects observed with the four combinations of hosts and parasites used and the range of conditions tested, we do not believe that other combinations would drastically change our results. However, we cannot exclude with certainty that some combinations might lead to a different result.

### Resistance or susceptibility of other *Daphnia *species

One clone of each of six other *Daphnia *species (*D. arenata*, *D. dolichocephala*, *D. galeata*, *D. barbata*, *D. similis *and *D. lumholtzi*) were assayed for their propensity of oesophageal spore attachment using *P. ramosa *clone C19. For this assay, groups of five conspecific individuals were exposed to 200,000 *P. ramosa *spores of clone C19 in 20 mL medium. Four replicates per species were used. After 5 days we filled the jars to 100 mL and then changed the medium on a weekly basis. Animals were fed daily with 5 to 10 million algal cells per jar depending on the size of the *Daphnia *species. The infection status was investigated under a microscope with phase contrast (magnification 400 ×), at host death or 29 days after exposure.

### Electron microscopy

In order to prepare *Daphnia *for transmission electron microscopy (TEM), infected individuals were fixed on ice in 4% glutaraldehyde buffer in Sorensen's phosphate buffer (0.1 M KH_2_PO_4 _and 0.1 M Na_2_HPO_4_) and kept in the dark for several hours. The animals were then rinsed five times on ice using the same buffer for a total of 5 min. Post-fixation was carried out with 1% OsO_4 _in Sorensen's phosphate buffer on ice. After post-fixation, the *Daphnia *were again washed in Sorensen's phosphate buffer on ice, dehydrated in a graded acetone series, and finally embedded in the epoxydic resin EPON.

Transversal and sagittal sections were made through the oesophagus. Semi-thin sections (diamond knife, 0.7-1 μm) were cut in order to approach the right spot on the resin block using a RMC MT 6000-XL (RMC Inc, AZ, USA) ultramicrotome. In order to identify regions of interest for TEM, the tissue was stained using Richardson's dye [57] and examined under a light microscope. In order to see parasite structures using TEM, 5-8 ultrathin sections (diamond knife, 60 nm) were cut after every 10 semi-thin sections. The ultra-thin sections were mounted on Formvar-coated copper grids and stained with uranyl acetate and lead citrate to enhance the contrast. Ultrathin sections were analysed using a FEI Morgagni™ transmission electron microscope at 80 kV equipped with a digital camera.

For scanning electron microscopy (SEM), *D. magna *were fixed in 3% glutaraldehyde buffer in 0.1 M phosphate buffer for 2 h at 20°C. Samples were washed twice in distilled water for 5 - 10 s, dehydrated in graded ethanol series and critical point dried overnight (16 h). The specimens were coated with gold (20 nm) and viewed using a Philips XL 30 ESEM under high volume conditions from 5 to 15 kv.

## Authors' contributions

DD conceived and designed the study, performed the experiment, performed data analysis and drafted the manuscript. PL participated in the design of the study. FB performed the SEM. CL performed the TEM. DE conceived of the study, participated in its design and participated in drafting the manuscript. All authors read and approved the final manuscript.
